# A failure criterion for genuinely orthotropic materials and integration of a series of criteria for materials of different degrees of anisotropy

**DOI:** 10.1098/rsos.240205

**Published:** 2024-05-22

**Authors:** Mingming Xu, Elena Sitnikova, Shuguang Li

**Affiliations:** Faculty of Engineering, University of Nottingham, Nottingham NG8 1BB, UK

**Keywords:** failure criterion, orthotropic materials, strength, quadratic failure function, quadric surface, Tsai–Wu criterion

## Abstract

Existing failure criteria for orthotropic materials are subject to an underlying assumption which cause contradictions when applied to genuinely orthotropic materials that are significantly anisotropic in elasticity as well as in strengths. For such materials, there is lack of consistent failure criteria to support their applications in engineering structures. A general quadratic failure criterion tends to leave undetermined coefficients for interactive terms. A rational approach is adopted in this paper based on mathematical and logical considerations to determine these coefficients as the objective of this paper. Considerations are based on the intrinsic characteristics of the quadric surfaces introduced by the quadratic failure criterion. These coefficients must take the values as obtained, leaving no alternatives if logic prevails. The obtained criterion integrates for the first time a range of criteria separately formulated for materials of different degrees of anisotropy, from genuinely orthotropic, through transversely isotropic, cubically symmetric, to completely isotropic ones with different or identical tensile and compression strengths.

## 1. Introduction

The formulation of a material failure criterion is an empirical exercise of constructing a failure function through which a failure envelope can be generated in the stress or strain space using practically obtainable material properties. The inevitable empiricism involved often leaves an undesirable legacy behind as if mathematical rigour is no longer relevant. In this paper, a case will be argued that while empiricism is necessary to set the scene for investigation, the subsequent development should and can be followed through rationally (Table 1).

**Table 1.** Nomenclature (listed according to their order of appearance in the paper).

F:	failure function
$\sigma_{11},\sigma_{22},\sigma_{33},\tau_{23},\tau_{13}$, and $\tau_{12}$:	stress components in the principal directions of the orthotropic material
$\sigma_{i}$:	same as $\sigma_{11},\sigma_{22},\sigma_{33},\tau_{23},\tau_{13}$, and $\tau_{12}$ but expressed in the contracted form
*F_i_*, *F_ij_* and *F_ijk_* (*i*, *j*, *k* = 1, 2, … 6):	coefficients to the 1st-, 2nd- and 3rd-order terms of stresses
*α*, *β* and *γ* :	power to the 1st-, 2nd- and 3rd-order terms of stresses
*F*, *G*, *H*, *L*, *M* and *N*:	coefficients in the quadratic function of stresses in the Hill criterion
*F*, *G*, *H*, *P*, *Q* and *R*:	coefficients to different stress combinations in the modified Hill criterion
*m*:	power to all terms of different stress combinations in the modified Hill criterion
*F*, *G*, *H*, *L*, *M*, *N*, *I*, *J* and *K*:	coefficients in the quadratic function of stresses in another modified Hill criterion
*F*, *G*, *H*, *L*, *M*, *N* and *S*:	coefficients in the quadratic function of stresses in the criterion of Deshpande *et al*.
*X*_t_, *X*_c_, *Y*_t_, *Y*_c_, *Z*_t_ and *Z*_c_:	tensile and compressive strengths of an orthotropic material in its principal directions
*S*_23_, *S*_13_ and *S*_12_:	shear strength of the orthotropic material in its principal planes
${\tilde{\sigma }}_{11},{\tilde{\sigma }}_{22},{\tilde{\sigma }}_{33},{\tilde{\tau }}_{23},{\tilde{\tau }}_{13}\;\mathrm{and}\;{\tilde{\tau }}_{12}$:	stresses normalized with respect to their respective strengths
${\tilde{F}}_{23}$, ${\tilde{F}}_{13}$, ${\tilde{F}}_{12}$, ${\tilde{F}}_{1}$, ${\tilde{F}}_{2}$ and ${\tilde{F}}_{3}$:	coefficients of the quadratic failure function normalized with respect to respective strengths
$K=1-({\tilde{\tau }}_{23}^{2}+{\tilde{\tau }}_{13}^{2}+{\tilde{\tau }}_{12}^{2})$:	effective right-hand side of the quadric surface for the failure envelope, having the contributions of the shear stresses absorbed in it
I, *J*, *A*, *A′* and *D* = det(**D**):	invariants of a quadric surface
D	coefficient matrix of the 2nd-order terms
$\lambda_{1}$ and $\lambda_{2,3}$:	1st and the 2nd and 3rd eigenvalues of **D**
φ:	intermediate function introduced to apply the Lagrangian multiplier
η:	Lagrangian multiplier
ξ=−(1−η)/η:	constant as an expression of the Lagrangian multiplier
${\left[\begin{array}{ccc} {\tilde{\sigma }}_{1} & {\tilde{\sigma }}_{2} & {\tilde{\sigma }}_{3} \end{array}\right]}^{T}$:	eigenvector of **D** associated with $\lambda_{1}$
$S_{T}$:	transverse shear strength of a transversely isotropic material
$X_{t},X_{c}\;\mathrm{and}\;S$:	tensile, compressive and shear strengths of an isotropic material
X:	strength of an isotropic material, equal under tension and compression
p and c:	coefficients in standardized form of quadric surfaces
${\left[\begin{array}{ccc} s_{11} & s_{22} & s_{33} \end{array}\right]}^{T}$:	coordinates for the standardized quadric surfaces
*t*_1_, *t*_2_ and *t*_3_:	principal directions of the quadric surface
$\delta_{1}$, $\delta_{2}$ and $\delta_{3}$:	translations required to standardize quadric surface

**Figure 1.**
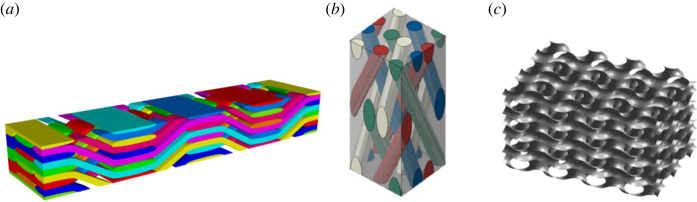
Examples of micro-/meso-structures giving rise to genuinely orthotropic materials whose principal axes were identified using the reflectional and/or rotational symmetries [8]. (*a*) A three-dimensional woven composite, (*b*) a three-dimensional braided composite, and (*c*) a gyroid lattice structure.

Anisotropic materials have been common in modern engineering due to the wide use of fibre reinforced composites. To date, however, most composites in structural applications are in the form of laminates composed of unidirectionally fibre reinforced laminae which are typically transversely isotropic. As a result, the most well-known composite material failure criteria are either formulated specifically for transversely isotropic materials, e.g. the Hashin criterion [1], the Puck criterion [2] and LaRC05 [3], to name but a few, or applied practically only to such materials, e.g. the Tsai–Wu criterion [4–7]. Except those to be reviewed in the next section, where most of them were designated for materials of minor anisotropy, there have not been many well-established failure criteria for genuinely orthotropic materials. Genuinely orthotropic materials are different from materials of minor anisotropy in two aspects: (i) the anisotropy involved is significant and (ii) the strength properties are as anisotropic as elastic properties. For instance, modern fibre reinforced composites are elastically anisotropic as much as in their strength characteristics, if not more. These can further be exacerbated by the significant disparity in strengths between tension and compression. Such materials are relatively emerging technology in terms of their practical applications, although their theoretical definition has been well known. Several examples as cited in [8] are illustrated in figure 1, which are being made possible with modern technological developments, such as modern composites reinforced with three-dimensional textile preforms and 3D printed lattice structures, potentially as structural or functional materials. Such materials are becoming more and more readily available, but their practical applications are very limited, the lack of appropriate design tools being one of the reasons, of which failure criteria are an important part. The lack of applications and the lack of design tools seem to form a chicken-and-egg scenario. It is hoped that the failure criterion presented in this paper would help to break this deadlock and therefore promote the acceptance of such materials in engineering and applications of them seriously.

On the subject of failure criteria for anisotropic materials, the state-of-the-art of the development can be briefly reviewed as follows.

One of the most comprehensive presentations of empiricism for anisotropic materials using a single failure function explicitly expressed in terms of stress was given as follows [9]:

1.1 $${\left(F_{i}\sigma_{i}\right)}^{\alpha }+{\left(F_{ij}\sigma_{i}\sigma_{j}\right)}^{\beta }+{\left(F_{ijk}\sigma_{i}\sigma_{j}\sigma_{k}\right)}^{\gamma }+\ldots =1,$$

where the contracted tensor notation is used, *σ_i_* being stress tensor, *F*'s various coefficients to be determined and *i*, *j*, *k* = 1, 2, … 6. There has never been a single attempt of failure prediction that went nearly as far as (1.1) and most took the first two terms with *α* = *β* = 1 [4] or even simpler combinations. Even so, users have been overwhelmed by the demand for the determination of all the coefficients involved. While some of the coefficients could be determined one way or another, some have been left loose to a great extent. Although (1.1) was supposed to be applicable to generally anisotropic materials under three-dimensional stress states, there have never been such materials in practical applications and no industrial standards are available to characterize such materials practically. In terms anisotropy, practicality allows materials to go as far as being orthotropic.

Existing accounts on failure criteria for orthotropic materials can be found in the literature (e.g. [10–16]). Hill was perhaps the first who proposed a yield criterion [10] for the plasticity of metals which exhibited a degree of anisotropy because they had been processed in certain ways, such as rolling. It was given as follows:

1.2 $$F{\left(\sigma_{22}-\sigma_{33}\right)}^{2}+G{\left(\sigma_{33}-\sigma_{11}\right)}^{2}+H{\left(\sigma_{11}-\sigma_{22}\right)}^{2}+2M\tau_{23}^{2}+2N\tau_{13}^{2}+2L\tau_{12}^{2}=1,$$

where *F*, *G*, *H*, *L*, *M* and *N* are coefficients in the quadratic function. The direct stresses were delicately presented in the form as given in (1.2) so that the yield surface will exhibit the following characteristics. In the subspace of direct stresses, it formed an elliptic cylinder. The axis of it follows the loading path under a hydrostatic stress condition. Infinite strengths for both tension and compression were expected under such a stress condition.

Hill subsequently modified his criterion into (under direct stresses only) [11]

1.3 $$\begin{array}{rl} F|\sigma_{22}-\sigma_{33}{|}^{m}+ & G|\sigma_{33}-\sigma_{11}{|}^{m}+H|\sigma_{11}-\sigma_{22}{|}^{m}\\ & \;+P|2\sigma_{11}-\sigma_{22}-\sigma_{33}{|}^{m}+Q|2\sigma_{22}-\sigma_{33}-\sigma_{11}{|}^{m}\\ & \;+R|2\sigma_{33}-\sigma_{11}-\sigma_{22}{|}^{m}=1, \end{array}$$

where *F*, *G*, *H*, *P*, *Q* and *R* are coefficients to be determined through experiments, along with parameter *m*. However, it was soon found in [12] that it could lead to impermissible behaviour, e.g. concave yield surface which was not acceptable for plasticity.

Hill's original criterion (1.2) was modified to accommodate the disparities in tensile and compressive strengths for orthotropic materials, leading to the following criterion [13]:

1.4 $$\begin{array}{rl} F{\left(\sigma_{22}-\sigma_{33}\right)}^{2}+G{\left(\sigma_{33}-\sigma_{11}\right)}^{2}+ & H{\left(\sigma_{11}-\sigma_{22}\right)}^{2}+2L\tau_{23}^{2}+2M\tau_{13}^{2}\\ & \;+2N\tau_{12}^{2}+I\sigma_{11}+J\sigma_{22}+K\sigma_{33}=1, \end{array}$$

with three new coefficients *I*, *J* and *K* introduced and to be determined by experiments. This turned the yield surface from an elliptic cylinder into an elliptic paraboloid.

Another alteration to the original Hill criterion was proposed for materials of lattice structures in [14] as

1.5 $$\begin{array}{rl} F{\left(\sigma_{22}-\sigma_{33}\right)}^{2}+G{\left(\sigma_{33}-\sigma_{11}\right)}^{2}+ & H{\left(\sigma_{11}-\sigma_{22}\right)}^{2}+2L\tau_{23}^{2}+2M\tau_{13}^{2}\\ & \;+2N\tau_{12}^{2}+S{\left(\sigma_{11}+\sigma_{22}+\sigma_{33}\right)}^{2}=1, \end{array}$$

with a new coefficient *S* to be determined by experiments. The term associated with it represents the contribution of hydrostatic stress to yield. The introduction of this term turned the yield surface into an ellipsoid, if *S* was positive as it should be. For porous materials, it is reasonable to expect finite strength under all stress conditions and hence a close ellipsoidal yield surface will be more appropriate.

In all above criteria, with the yield surfaces being elliptic cylinder, elliptic paraboloid and ellipsoid, they share a common feature that the axis (one of the axes in the case of ellipsoid) is orientated at equal angle to each of the three axes of direct stresses. In the cases of elliptic cylinder, infinite strengths are expected under both hydrostatic tension and compression, whereas with elliptic paraboloid, infinite strength is only possible under hydrostatic compression.

In [15] as another development, an elliptic paraboloidal failure envelope was employed, leading to a failure surface effectively the same as (1.4). It employed an interesting description for the consequence of the infinite strength under hydrostatic compression as the symmetry between different direct stresses, which is questionable as will be elaborated later.

The failure criterion found in [16] was in fact identical to what will be achieved in this paper. However, it came as a pure postulation in [16] without any reference or justification.

Infinite strength under hydrostatic compression is well justified and well accepted for isotropic solid materials. It was the assumption on which the von Mises criterion [17] was established. In fact, the same assumption was also implied in the Tresca criterion [18]. Physically, an infinite strength is a position that can never be experimentally validated. The study of metal plasticity owed greatly to Bridgman's systematic experiments [19,20] conducted in the presence of extremely high hydrostatic pressures where no noticeable plastic deformation was observed. He won his Noble prize for his work in the field involving such high pressures. Infinite strength under hydrostatic pressure was thus postulated for metals. For isotropic materials, the deformation generated by a hydrostatic stress is a dilatational strain state. Allowance of infinite strength under hydrostatic stress is equivalent to allowance of infinite strength under dilatational strain. This correspondence does not hold for anisotropic materials anymore in general, because hydrostatic stress results in distortional strains and dilatational strain comes with deviatoric stresses. Infinite hydrostatic stress or dilatational strain will imply unlimited distortional strain or unlimited deviatoric stress, respectively, neither supporting infinite strength reasonably without causing contradiction with common sense. Even if one is prepared to accept a blind extension from isotropic materials to anisotropic ones, one would be left with a dilemma between infinite strength under hydrostatic stress or that under dilatational deformation.

It is important to point out that all criteria as presented in [10–13,15] were so formulated that their applications were made primarily to materials of minor anisotropy. In particular, the anisotropy was mostly present in the strengths of the material but not so much in its elasticity. In this case, a hydrostatic stress state still coincides more or less with a dilatational strain state. For genuinely orthotropic materials, a hydrostatic stress state and a dilatational strain state are usually far apart. This leaves a gap in the development of this important field.

It is also of interest to notice that hydrostatic stress and dilatational strain are both descriptors of elastic deformations. They coincide with each other conveniently for isotropic materials and therefore they cause no conflict while offering a useful condition to eliminate some constants to be determined otherwise. None of them would offer consistent justification for infinite strength anymore without a fundamental contradiction for genuinely orthotropic materials, in general, because at an increasing loading, a hydrostatic stress state will generate excessive distortional strains, while a dilatational strain state is accompanied with excessive deviatoric stresses. None of them is acceptable in the justification of infinite strength in the usual way for isotropic materials. The reality is that there is lack of experimental data about the true behaviour of such materials under these stress or strain states. Until such information becomes available, a compromise is to leave the elastic properties aside and to construct the failure criterion purely based on the strength properties, as will be pursued in this paper. Undoubtedly, this is not expected to deliver a comprehensive and universally applicable theory. No such theory is possible in any foreseeable future, no matter how many aspects have been taken into consideration and how sophisticated the theory has become. As a modest objective, this paper will focus on a failure criterion formulated as simple as possible in its final presentation while maintaining a basic level of consistency within its predefined realm. The failure envelope will be classified into a definitive form of quadric surface, while most existing criteria tended to shy away from an explicit description of the shape of their failure envelope in space, since a hexagonal or a circular cylinder was explicitly specified in the Tresca and the von Mises criteria, respectively. For the present development, the criterion is to be constructed using a single quadratic failure function based on strength considerations only, while the elastic deformation will be kept aside.

An embarrassment of a simple criterion as stated above is that, on the one hand, there is a shortage of conditions available to evaluate some of the constants to be determined, but on the other hand, conditions associated with elastic deformation cannot be used in the formulation of the failure criterion. This is unfortunately the cost of the modesty in the objective to be achieved. Even so, making an effort to achieve it is still worthwhile as it will ease the resistance to wide acceptance of genuinely orthotropic materials in engineering.

Under the restriction that materials concerned are transversely isotropic as a special type of orthotropy, a fully rational failure criterion has been achieved [6,7] under three-dimensional stress states. However, for genuinely orthotropic materials under a three-dimensional stress condition, such rationalization is still yet to be established, which is the subject of the present paper.

To deliver the failure criterion to be established, a systematic approach will be taken in this paper. A quadratic failure function of stresses will be mathematically presented and normalized for the subsequent development in the next section. It will be argued that the failure criterion generated from the quadratic function can be represented in a three-dimensional subspace of direct stresses only, in which the characteristics of the failure criterion as a quadric surface can be fully determined in terms of a limited number of invariants which are well defined in analytic geometry. This will be described clearly in §3 and it will also be argued there that the acceptable failure envelope can only be a paraboloid, in general. In §4, the mathematical implication of the failure envelope being a paraboloid will be explored logically. This eventually leads to the determination of the coefficients to the interactive terms between direct stresses in §5, delivering the failure criterion to be formulated as a result. This failure criterion is then integrated with a family of existing failure criteria as special cases of the formulated criterion, as achieved in §6. An attempt will be made in §7 to transform the failure envelope for the formulated criterion into a standard form of quadric surface to highlight the characteristics of the failure envelope. A number of aspects are discussed in §8, before relevant conclusions are drawn in §9.

## 2. A quadratic failure function and relevant normalization

The classical quadratic failure function was proposed for orthotropic materials in [4] as a concession from its formidable form (1.1) as

2.1 $$F=F_{i}\sigma_{i}+F_{ij}\sigma_{i}\sigma_{j}.$$

The coefficients to second order terms are symmetric, i.e. $F_{ij}=F_{ji}$. Failure function (2.1) is defined in the 6-dimensional stress space, in general. Due to the symmetries present in orthotropic materials in general, if the coordinate axes are chosen to coincide with the principal axes of orthotropy, terms involving an odd order of any shear stress component are deemed to vanish. The failure criterion for orthotropic materials in the terms of conventional stress components can be written as

2.2a F=1,

or

2.2b $$\begin{array}{r} F_{11}\sigma_{11}^{2}+F_{22}\sigma_{22}^{2}+F_{33}\sigma_{33}^{2}+2F_{12}\sigma_{11}\sigma_{22}+2F_{13}\sigma_{11}\sigma_{33}+2F_{23}\sigma_{22}\sigma_{33}\\ +F_{1}\sigma_{11}+F_{2}\sigma_{22}+F_{3}\sigma_{33}+F_{44}\tau_{23}^{2}+F_{55}\tau_{13}^{2}+F_{66}\tau_{12}^{2}=1. \end{array}$$

For this criterion to reproduce the input data, some of the coefficients in (2.2*b*) must take the following forms:

2.3a $$F_{1}=\frac{1}{X_{t}}-\frac{1}{X_{c}},F_{2}=\frac{1}{Y_{t}}-\frac{1}{Y_{c}},F_{3}=\frac{1}{Z_{t}}-\frac{1}{Z_{c}},$$

2.3b $$F_{11}=\frac{1}{X_{t}X_{c}},F_{22}=\frac{1}{Y_{t}Y_{c}},F_{33}=\frac{1}{Z_{t}Z_{c}},$$

2.3c $$F_{44}=\frac{1}{S_{23}^{2}},F_{55}=\frac{1}{S_{13}^{2}}\;\mathrm{and}\;F_{66}=\frac{1}{S_{12}^{2}},$$

where *X_t_*, *X_c_*, *Y_t_*, *Y_c_*, *Z_t_* and *Z_c_* are the tensile and compressive strengths of the material in its principal directions obtained under uniaxial stress states, and *S*_23_, *S*_13_ and *S*_12_ are the shear strength of the material in its principal planes obtained under pure shear stress states. These are measurable strength properties of the material, that can be employed as the input data available to users.

The remaining coefficients, *F*_23_, *F*_13_ and *F*_12_, are associated with the interactive terms between direct stresses. There has never been any convincing means to measure them experimentally. They have been dropped from the failure function in some accounts or artificially prescribed values in others, giving rise to various types of different failure criteria. In [10–13,15], they were determined under the condition that the material exhibits infinite strength when it is subjected to hydrostatic compression, as was mentioned in the previous section.

Given the anisotropy of the material under consideration, stress components at failure are normalized with respect to the respective strengths as follows so that their contributions to failure can be evaluated on a common basis:

2.4 $$\begin{array}{c} {\tilde{\sigma }}_{11}=\sqrt{F_{11}}\sigma_{11}\\ {\tilde{\sigma }}_{22}=\sqrt{F_{22}}\sigma_{22}\\ {\tilde{\sigma }}_{33}=\sqrt{F_{33}}\sigma_{33} \end{array}\;\mathrm{and}\;\begin{array}{c} {\tilde{\tau }}_{23}=\sqrt{F_{44}}\tau_{23}\\ {\tilde{\tau }}_{13}=\sqrt{F_{55}}\tau_{13}\\ \;{\tilde{\tau }}_{12}=\sqrt{F_{66}}\tau_{12}. \end{array}$$

Accordingly, criterion (2.2) is normalized into

2.5 $$\begin{array}{rl} & {\tilde{\sigma }}_{11}^{2}+{\tilde{\sigma }}_{22}^{2}+{\tilde{\sigma }}_{33}^{2}+2{\tilde{F}}_{23}{\tilde{\sigma }}_{22}{\tilde{\sigma }}_{33}+2{\tilde{F}}_{13}{\tilde{\sigma }}_{33}{\tilde{\sigma }}_{11}+2{\tilde{F}}_{12}{\tilde{\sigma }}_{11}{\tilde{\sigma }}_{22}\\ & \;+{\tilde{F}}_{1}{\tilde{\sigma }}_{11}+{\tilde{F}}_{2}{\tilde{\sigma }}_{22}+{\tilde{F}}_{3}{\tilde{\sigma }}_{33}+{\tilde{\tau }}_{23}^{2}+{\tilde{\tau }}_{13}^{2}+{\tilde{\tau }}_{12}^{2}=1, \end{array}$$

where

2.6 $$\begin{array}{rl} {\tilde{F}}_{23}= & \frac{F_{23}}{\sqrt{F_{22}F_{33}}},\\ {\tilde{F}}_{13}= & \frac{F_{13}}{\sqrt{F_{11}F_{33}}},\\ {\tilde{F}}_{12}= & \frac{F_{12}}{\sqrt{F_{11}F_{22}}},\\ {\tilde{F}}_{1}= & \frac{F_{1}}{\sqrt{F_{11}}},\\ {\tilde{F}}_{2}= & \frac{F_{2}}{\sqrt{F_{22}}}\\ \mathrm{and}\;{\tilde{F}}_{3}= & \frac{F_{3}}{\sqrt{F_{33}}}. \end{array}\}$$

The direct consequence of the normalization is that the anisotropy has been harmonized to a great extent, such that the squared terms of stresses share the common coefficient of unity. However, the harmonization so far is only partial, since the coefficients to the interactive terms could still be different. A complete harmonization will be achieved after these coefficients have been determined later. It should be noted that the factors employed in the above normalization are expressions of known strength properties as involved in (2.3).

Before proceeding any further, it is helpful to impose the following constraints to the interactive terms:

2.7 $${\tilde{F}}_{ij}^{2}<1\;\;i,j=1,2,3\;\mathrm{and}\;i\neq j.$$

These restrictions were initially introduced by Tsai & Wu [4] (with ≤ replaced by <) as conditions for a closed failure envelope but their precise implication based on analytic geometry was only made completely clear in [5–7], i.e. as both necessary and sufficient conditions for the intersections of the failure envelope in the three-dimensional subspace of direct stresses with three coordinate planes to be ellipses and hence closed. These conditions can be justified as follows. There has been sufficiently large number of test data, published or not, about the strengths of existing composites or other materials under two-dimensional stress states, and infinite strength has never been observed or suggested. There is good reason to believe that no infinite strength is possible when an orthotropic material is under a plane stress state in any of its principal planes. Of course, this does not exclude the possibility of infinite strength under a three-dimensional stress condition.

Constraints (2.7) are not sufficient to deliver a closed failure envelope, in general. A closed failure envelope is, in fact, not desirable as it leads to difficulties when isotropic materials of equal tensile and compressive strengths are considered as a special case of orthotropic materials. For them, the von Mises criterion [17] is expected to apply. The failure envelope then is a cylinder and hence is open. A generally applicable failure envelope will have to be open somehow, allowing an infinite strength under a certain stress condition. An infinite strength for isotropic metals was justified by Bridgman's high pressure experiments [19] to a degree. For composites, no one has ever tested them at pressures anywhere near what Bridgman did. As a result, postulating an infinite strength has never been a comfortable position in the development of failure criteria for composites. On the other hand, claiming a finite strength under any stress condition is probably an even weaker position to defend, if one wishes to embrace the von Mises criterion as a special case. The compromise is to minimize the opening of the failure envelope so that infinite strength is only allowed for a single stress condition.

## 3. The three-dimensional subspace of direct stresses and its characteristics

For the ease of formulating the failure criterion, the stress space can be reduced from a general 6-dimensional one including the shear stresses to a three-dimensional subspace involving only direct stresses. This is possible because the appearances of shear stresses in the failure function (2.5) are all in full square form without any interaction with any other stress components or each other. Each shear stress component makes positive contribution to the failure monotonically. Their effects can be fully incorporated as a reduction of the right-hand side of (2.5) from 1 to a lower value as argued in [5–7]. Denoting

3.1 $$K=1-({\tilde{\tau }}_{23}^{2}+{\tilde{\tau }}_{13}^{2}+{\tilde{\tau }}_{12}^{2}),$$

the failure criterion (2.5) can be rewritten as

3.2 $$\begin{array}{r} {\tilde{\sigma }}_{11}^{2}+{\tilde{\sigma }}_{22}^{2}+{\tilde{\sigma }}_{33}^{2}+2{\tilde{F}}_{23}{\tilde{\sigma }}_{22}{\tilde{\sigma }}_{33}+2{\tilde{F}}_{13}{\tilde{\sigma }}_{33}{\tilde{\sigma }}_{11}+2{\tilde{F}}_{12}{\tilde{\sigma }}_{11}{\tilde{\sigma }}_{22}\\ +{\tilde{F}}_{1}{\tilde{\sigma }}_{11}+{\tilde{F}}_{2}{\tilde{\sigma }}_{22}+{\tilde{F}}_{3}{\tilde{\sigma }}_{33}-K=0. \end{array}$$

In the presence of shear stresses, constant *K* could be negative as well as positive, but definitely less than 1. Thus, the investigation of the failure function can be confined within the subspace of the three direct stresses.

In the direct stress space, the failure criterion (3.2) defines a quadric surface which involves only three coefficients to be determined. They are associated with the interactive terms between the direct stresses. Unfortunately, there has been no established approach to determine any of them experimentally. Alternative ways may have to be explored to bring forward a convincing solution. Readers are reminded that *K* is not an unknown as it is fully determined once the shear stresses are given although it involves three shear strengths as expressed in (2.3*c*).

According to analytic geometry [21], the characteristics of a quadric surface in the ${\tilde{\sigma }}_{11}\text{–}{\tilde{\sigma }}_{22}\text{–}{\tilde{\sigma }}_{33}$ three-dimensional space as given in (3.2), can be completely determined by the following invariants:

3.3a I=1+1+1=3>0given (2.7),

3.3b $$J=\left|\begin{array}{cc} 1 & {\tilde{F}}_{12}\\ {\tilde{F}}_{12} & 1 \end{array}\right|+\left|\begin{array}{cc} 1 & {\tilde{F}}_{23}\\ {\tilde{F}}_{23} & 1 \end{array}\right|+\left|\begin{array}{cc} 1 & {\tilde{F}}_{13}\\ {\tilde{F}}_{13} & 1 \end{array}\right|=3-{\tilde{F}}_{12}^{2}-{\tilde{F}}_{13}^{2}-{\tilde{F}}_{23}^{2}>0,$$

3.3c $$D=|D|=1-{\tilde{F}}_{12}^{2}-{\tilde{F}}_{13}^{2}-{\tilde{F}}_{23}^{2}+2{\tilde{F}}_{12}{\tilde{F}}_{13}{\tilde{F}}_{23}\;\mathrm{with}\;D=\left[\begin{array}{ccc} 1 & {\tilde{F}}_{12} & {\tilde{F}}_{13}\\ {\tilde{F}}_{12} & 1 & {\tilde{F}}_{23}\\ {\tilde{F}}_{13} & {\tilde{F}}_{23} & 1 \end{array}\right],$$

3.3d $$A=\left|\begin{array}{cccc} 1 & {\tilde{F}}_{12} & {\tilde{F}}_{13} & \frac{1}{2}{\tilde{F}}_{1}\\ {\tilde{F}}_{12} & 1 & {\tilde{F}}_{23} & \frac{1}{2}{\tilde{F}}_{2}\\ {\tilde{F}}_{13} & {\tilde{F}}_{23} & 1 & \frac{1}{2}{\tilde{F}}_{3}\\ \frac{1}{2}{\tilde{F}}_{1} & \frac{1}{2}{\tilde{F}}_{2} & \frac{1}{2}{\tilde{F}}_{3} & -K \end{array}\right|$$

3.3e $$\mathrm{and}\;A^{\prime }=\left|\begin{array}{ccc} 1 & {\tilde{F}}_{23} & \frac{1}{2}{\tilde{F}}_{2}\\ {\tilde{F}}_{23} & 1 & \frac{1}{2}{\tilde{F}}_{3}\\ \frac{1}{2}{\tilde{F}}_{2} & \frac{1}{2}{\tilde{F}}_{3} & -K \end{array}\right|+\left|\begin{array}{ccc} 1 & {\tilde{F}}_{13} & \frac{1}{2}{\tilde{F}}_{1}\\ {\tilde{F}}_{13} & 1 & \frac{1}{2}{\tilde{F}}_{3}\\ \frac{1}{2}{\tilde{F}}_{1} & \frac{1}{2}{\tilde{F}}_{3} & -K \end{array}\right|+\left|\begin{array}{ccc} 1 & {\tilde{F}}_{12} & \frac{1}{2}{\tilde{F}}_{1}\\ {\tilde{F}}_{12} & 1 & \frac{1}{2}{\tilde{F}}_{2}\\ \frac{1}{2}{\tilde{F}}_{1} & \frac{1}{2}{\tilde{F}}_{2} & -K \end{array}\right|+\left|\begin{array}{ccc} 1 & {\tilde{F}}_{12} & {\tilde{F}}_{13}\\ {\tilde{F}}_{12} & 1 & {\tilde{F}}_{23}\\ {\tilde{F}}_{13} & {\tilde{F}}_{23} & 1 \end{array}\right|.$$

With the definitive sense of *I* and *J* as shown in (3.3*a*) and (3.3*b*), a real surface can be obtained under the following conditions [21], while other combinations will lead to lines, points or even imaginary objects:

3.4a $$\mathrm{If}\;A\;<0\;(\mathrm{any}\;A^{\prime })\;\mathrm{and}\;D\;>\;0,\;\mathrm{it}\;\mathrm{is}\;\mathrm{an}\;\mathrm{ellipsoid};$$

3.4b $$\mathrm{if}\;A<0\;(\mathrm{any}\;A^{\prime })\;\mathrm{and}\;D=0,\;\mathrm{it}\;\mathrm{is}\;\mathrm{an}\;\mathrm{elliptic}\;\mathrm{paraboloid};$$

3.4c $$\mathrm{if}\;A<0\;(\mathrm{any}\;A^{\prime })\;\mathrm{and}\;D<0,\;\mathrm{it}\;\mathrm{is}\;a\;\text{two-sheets}\;\mathrm{hyperboloid};$$

3.4d $$\mathrm{if}\;A>0,\;A^{\prime }<0\;\mathrm{and}\;D<0,\;\mathrm{it}\;\mathrm{is}\;a\;\text{one-sheet}\;\mathrm{hyperboloid};$$

3.4e $$\mathrm{and}\;\mathrm{if}\;A=0,A^{\prime }<0\;\mathrm{and}\;D=0,\;\mathrm{it}\;\mathrm{is}\;\mathrm{an}\;\mathrm{elliptic}\;\mathrm{cylinder}.$$

Out of these surfaces, both types of hyperboloids can be ruled out straight away as suitable candidates for the failure envelope, as they allow infinite number of different stress ratios to exhibit infinite strength, given the existence of asymptotes as a character of hyperboloids in general. An ellipsoid would not allow infinite strength under any stress ratio, which would prevent the criterion to be reduced to the von Mises criterion if the material is isotropic and of equal tensile and compressive strengths. Surviving ones are an elliptic paraboloid and an elliptic cylinder and they are the available candidates for the failure envelope. Both share the same character of *D* = 0 and have a characteristic longitudinal axis and an elliptic cross-section. A cylindrical failure envelope is suitable if the tensile and compressive strengths are identical, the von Mises criterion being a special case when the material is isotropic as well. Otherwise, when tensile and compressive strengths are different, an elliptic paraboloid will be the only available option. The limitation on the availability of acceptable candidates is due to the use of a quadratic failure function. To break this limitation, one could go for failure functions of higher orders, as suggested through (1.1), or define the failure envelope in a piece-wise manner. While these will be interesting developments in future, they are beyond the scope of the present paper.

Given the expression of *D* as in (3.3*c*), *D* = 0 provides a condition among ${\tilde{F}}_{23}$, ${\tilde{F}}_{13}$ and ${\tilde{F}}_{12}$. Out of these three coefficients to the interactive terms in the failure criterion, only two are independent. While this eliminates one of these three troublesome coefficients, there are still two more to be determined.

## 4. The consequence of condition *D* = 0

**Figure 2.**
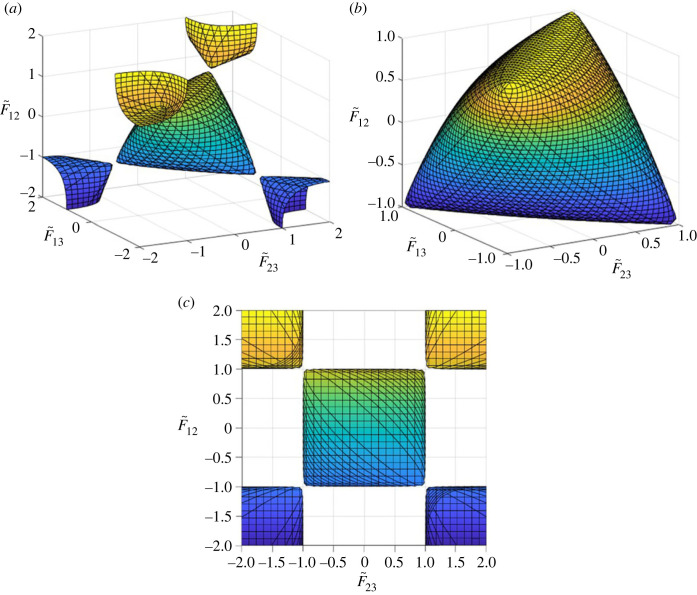
Surface *D* = 0 in the $\left({\tilde{F}}_{23},{\tilde{F}}_{13},{\tilde{F}}_{12}\right)$ space: (*a*) viewed at an angle; (*b*) the central rounded tetrahedron surface; (*c*) the direct view from the ${\tilde{F}}_{13}$ direction.

Consider condition *D* = 0 as a relationship among ${\tilde{F}}_{23}$, ${\tilde{F}}_{13}$ and ${\tilde{F}}_{12}$. Then it defines a surface in the ${\tilde{F}}_{23}\text{–}{\tilde{F}}_{13}\text{–}{\tilde{F}}_{12}$ space, which is shown in figure 2*a*. It can be considered as 5 almost disconnected pieces, 4 open lobes in the 1st, 3rd, 6th and 8th octants, respectively, where ${\tilde{F}}_{23}{\tilde{F}}_{13}{\tilde{F}}_{12}>0$, and one closed surface in the centre as shown in figure 2*b*, which looks like a rounded right tetrahedron with vertices located at $|{\tilde{F}}_{23}|=|{\tilde{F}}_{13}|=|{\tilde{F}}_{12}|=1$ in the 1st, 3rd, 6th and 8th octants, respectively, connected with the open lobe in the octant at a single point at its vertex there. At any point on any of the four open lobes, condition (2.7) is violated as can be visualized from figure 2*c* where a direct view of the *D* = 0 surface is shown, viewing along the direction of the ${\tilde{F}}_{13}$ axis. Such a planar view is in fact identical for any of the three coordinate directions. Since these four open lobes all violate (2.7), they can therefore be dismissed, leaving only the central rounded tetrahedron shown in figure 2*b* as the only piece of the surface on which permissible values of ${\tilde{F}}_{23}$, ${\tilde{F}}_{13}$ or ${\tilde{F}}_{12}$ should be obtained. Its six edges defined by $|{\tilde{F}}_{23}|=1$, $|{\tilde{F}}_{13}|=1$ or $|{\tilde{F}}_{12}|=1$, including four vertices can also be dismissed straight away as impermissible combinations of ${\tilde{F}}_{23}$, ${\tilde{F}}_{13}$ and ${\tilde{F}}_{12}$, since they violate (2.7).

## 5. Determination of ${\tilde{F}}_{23}$, ${\tilde{F}}_{13}$ and ${\tilde{F}}_{12}$ and its physical implications

As mentioned already, condition *D* = 0 comes with the presence of a longitudinal axis of the failure envelope, which is usually inclined at different angles to the coordinate axes, ${\tilde{\sigma }}_{11}$, ${\tilde{\sigma }}_{22}$ or ${\tilde{\sigma }}_{33}$, depending on the values of ${\tilde{F}}_{23}$, ${\tilde{F}}_{13}$ and ${\tilde{F}}_{12}$. The cross-section of the quadric surface is an ellipse in general and the aspect ratio of the ellipse also varies with the values of ${\tilde{F}}_{23}$, ${\tilde{F}}_{13}$ and ${\tilde{F}}_{12}$. The orientation of the axis and the aspect ratio of the cross-section are two important characteristics of the quadric surface. If there exists a condition under which the inclination of the longitudinal axis becomes unbiased among coordinate axes and the cross-section turns to circular at the same time, it should be interesting enough purely from the perspective of analytic geometry, let alone its physical significance. The mathematical condition to deliver such coincidence will be explored first before its physical implications are revealed.

The principal axes of a quadric surface (3.2) in the ${\tilde{\sigma }}_{11}\text{–}{\tilde{\sigma }}_{22}\text{–}{\tilde{\sigma }}_{33}$ space is defined by the eigenvectors of **D** matrix (3.3*c*) in general. A quadric surface can be analytically expressed in its standard form in such principal axes. Given *D* = |**D**| = 0, one of the eigenvalues must 0, i.e. $\lambda_{1}=0$. The eigenvector associated with this eigenvalue defined the orientation of the longitudinal axis.

The remaining two eigenvalues can be obtained as

5.1 $$\lambda_{2,3}=\frac{3\pm \sqrt{4({\tilde{F}}_{23}^{2}+{\tilde{F}}_{13}^{2}+{\tilde{F}}_{12}^{2})-3}}{2}.$$

For ${\tilde{F}}_{23}$, ${\tilde{F}}_{13}$ and ${\tilde{F}}_{12}$ to satisfy condition *D* = 0 and constraints (2.7), they can only take values on the surface as shown in figure 2*b*. Given the symmetry of matrix **D**, all eigenvalues of it should be real. This offers another condition for these to be determined coefficients, i.e. they have to be of sufficiently large absolute values to keep the expression inside the square root sign in (5.1) non-negative while satisfying constraints (2.7). It is therefore informative to find the minimum value of ${\tilde{F}}_{23}^{2}+{\tilde{F}}_{13}^{2}+{\tilde{F}}_{12}^{2}$ subject to condition *D* = 0. This can be achieved by introducing the following function, given the expression of *D* in (3.3*c*):

5.2 $$\varphi ={\tilde{F}}_{23}^{2}+{\tilde{F}}_{13}^{2}+{\tilde{F}}_{12}^{2}+\eta (1-{\tilde{F}}_{23}^{2}-{\tilde{F}}_{13}^{2}-{\tilde{F}}_{12}^{2}+2{\tilde{F}}_{23}{\tilde{F}}_{13}{\tilde{F}}_{12}),$$

with *η* being a Lagrangian multiplier to impose condition *D* = 0. The conditions for *φ* to take its stationary value are

5.3 $$\begin{array}{rl} & \frac{\partial \varphi }{\partial {\tilde{F}}_{23}}=2(1-\eta ){\tilde{F}}_{23}+2\eta {\tilde{F}}_{12}{\tilde{F}}_{13}=0\\ & \frac{\partial \varphi }{\partial {\tilde{F}}_{13}}=2(1-\eta ){\tilde{F}}_{13}+2\eta {\tilde{F}}_{12}{\tilde{F}}_{23}=0\\ & \frac{\partial \varphi }{\partial {\tilde{F}}_{12}}=2(1-\eta ){\tilde{F}}_{12}+2\eta {\tilde{F}}_{13}{\tilde{F}}_{23}=0. \end{array}$$

Adding any two of the three equations together leads to

5.4 $$\begin{array}{rl} & ((1-\eta )+\eta {\tilde{F}}_{23})({\tilde{F}}_{12}-{\tilde{F}}_{13})=0\\ & ((1-\eta )+\eta {\tilde{F}}_{12})({\tilde{F}}_{13}-{\tilde{F}}_{23})=0\\ & ((1-\eta )+\eta {\tilde{F}}_{13})({\tilde{F}}_{12}-{\tilde{F}}_{23})=0, \end{array}$$

giving an equivalent set of equations to (5.3), although only two of the above three equations are independent. All possible solutions to (5.4) can be obtained as

5.5 $${\tilde{F}}_{23}={\tilde{F}}_{13}={\tilde{F}}_{12}=-\frac{\left(1-\eta \right)}{\eta }\;\;\mathrm{from}\;\mathrm{the}\;\mathrm{first}\;\mathrm{factor}\;\mathrm{of}\;\mathrm{each}\;\mathrm{of}\;(5.4),$$

or

5.6 $${\tilde{F}}_{23}={\tilde{F}}_{13}={\tilde{F}}_{12}=\xi \;\mathrm{from}\;\mathrm{the}\;\mathrm{second}\;\mathrm{factor}\;\mathrm{of}\;\mathrm{each}\;\mathrm{of}\;(5.4),$$

where *ξ* is an arbitrary constant of an absolute value less than 1 in order to meet constraints (2.7). Apparently, no contradiction is present between (5.5) and (5.6), provided that *η* takes an appropriate value satisfying

5.7 $$\xi =-\frac{\left(1-\eta \right)}{\eta }.$$

In order to determine *ξ*, substituting (5.6) into condition *D* = 0, one has

5.8 $$1-{\tilde{F}}_{23}^{2}-{\tilde{F}}_{13}^{2}-{\tilde{F}}_{12}^{2}+2{\tilde{F}}_{12}{\tilde{F}}_{13}{\tilde{F}}_{23}=1-3\xi^{2}+2\xi^{3}=(1+2\xi ){\left(1-\xi \right)}^{2}=0.$$

All roots can be obtained as

5.9 $$\xi_{1}=-\frac{1}{2}\;\mathrm{and}\;\xi_{2,3}=1.$$

The latter repeats twice and it can be dismissed because of constraints (2.7). The only meaningful root is therefore the former which leads to

5.10 $${\tilde{F}}_{23}={\tilde{F}}_{13}={\tilde{F}}_{12}=\xi_{1}=-\frac{1}{2}.$$

With ${\tilde{F}}_{23}$, ${\tilde{F}}_{13}$ and ${\tilde{F}}_{12}$ determined, the value of *η* can also be determined from (5.7) as *η* = 2/3 which bears no significance.

Coefficients ${\tilde{F}}_{23}$, ${\tilde{F}}_{13}$ and ${\tilde{F}}_{12}$ as determined above define a unique point in the $\left({\tilde{F}}_{23},{\tilde{F}}_{13},{\tilde{F}}_{12}\right)$ space on surface *D* = 0, at which ${\tilde{F}}_{23}^{2}+{\tilde{F}}_{13}^{2}+{\tilde{F}}_{12}^{2}$ takes its minimum of 3/4. In this case, the two non-zero eigenvalues of **D** as given in (5.1) become identical, equal to 3/2, and therefore the cross-section of the quadric surface will be circular. Any two mutually perpendicular unit vectors in the plane perpendicular to the longitudinal axis will be the corresponding eigenvectors.

With ${\tilde{F}}_{23}$, ${\tilde{F}}_{13}$ and ${\tilde{F}}_{12}$ as determined above, the eigenvector corresponding to the eigenvalue $\lambda_{1}=0$ can be easily found as

5.11 $${\left[\begin{array}{ccc} {\tilde{\sigma }}_{1} & {\tilde{\sigma }}_{2} & {\tilde{\sigma }}_{3} \end{array}\right]}^{T}=\;\frac{1}{\sqrt{3}}{\left[\begin{array}{ccc} 1 & 1 & 1 \end{array}\right]}^{T}.$$

This defines the longitudinal axis of the quadric surface, which is apparently not biased towards any of the ${\tilde{\sigma }}_{11}$, ${\tilde{\sigma }}_{22}$ and ${\tilde{\sigma }}_{33}$ axes in the normalized direct stress space. The two distinct features of the quadric surface as described earlier synchronises. Such a coincidence and the sheer uniqueness of the set of values of ${\tilde{F}}_{23}$, ${\tilde{F}}_{13}$ and ${\tilde{F}}_{12}$ corresponding to it can only be taken as a strong hint from the nature signifying a fact that should not be ignored.

With ${\tilde{F}}_{23}$, ${\tilde{F}}_{13}$ and ${\tilde{F}}_{12}$ so determined, failure envelope (3.2) becomes

5.12a $${\tilde{\sigma }}_{11}^{2}+{\tilde{\sigma }}_{22}^{2}+{\tilde{\sigma }}_{33}^{2}-{\tilde{\sigma }}_{22}{\tilde{\sigma }}_{33}-{\tilde{\sigma }}_{33}{\tilde{\sigma }}_{11}-{\tilde{\sigma }}_{11}{\tilde{\sigma }}_{22}+{\tilde{F}}_{1}{\tilde{\sigma }}_{11}+{\tilde{F}}_{2}{\tilde{\sigma }}_{22}+{\tilde{F}}_{3}{\tilde{\sigma }}_{33}-K=0$$

or

5.12b $${\left({\tilde{\sigma }}_{22}-{\tilde{\sigma }}_{33}\right)}^{2}+{\left({\tilde{\sigma }}_{33}-{\tilde{\sigma }}_{11}\right)}^{2}+{\left({\tilde{\sigma }}_{11}-{\tilde{\sigma }}_{22}\right)}^{2}+2({\tilde{F}}_{1}{\tilde{\sigma }}_{11}+{\tilde{F}}_{2}{\tilde{\sigma }}_{22}+{\tilde{F}}_{3}{\tilde{\sigma }}_{33}-K)=0.$$

The perfect symmetry between any of the two normalized stresses among all quadratic terms has been achieved. This seems to resemble the symmetry postulation introduced in [15]. However, a fundamental difference is that the symmetry observed here is present only in the normalized stress space. If one returns to the conventional form before normalization, the longitudinal axis of the quadric surface will be biased according to the degree of anisotropy of the material and its cross-section will be a genuine ellipse, while maintaining the topology of the failure envelope in its normalized form.

Apart from the interesting features of quadric surface (5.12) from the perspective of analytic geometry, as a failure envelope, it bears the following physical implications, which should justify the acceptance of the failure criterion as obtained.

(a) The formulated failure criterion allows a perfect harmonization among normalized direct stresses and its form as given in (5.12) does not contain any unknown coefficients to be determined. Criterion (5.12) resembles the well-known RCY criterion [22] for isotropic materials if stresses are similarly normalized in the RCY criterion. The RCY criterion naturally degenerates to the von Mises criterion [17] if the tensile and compressive strengths happen to be the same.
(b) Consider *D* as a function of ${\tilde{F}}_{23}$, ${\tilde{F}}_{13}$ and ${\tilde{F}}_{12}$. The rounded tetrahedron in the ${\tilde{F}}_{23}\text{–}{\tilde{F}}_{13}\text{–}{\tilde{F}}_{12}$ space as shown in figure 2*b* represents condition *D* = 0. At point ${\tilde{F}}_{23}={\tilde{F}}_{13}={\tilde{F}}_{12}=-1/2$, *D* as a function of ${\tilde{F}}_{23}$, ${\tilde{F}}_{13}$ and ${\tilde{F}}_{12}$ is equally sensitive to ${\tilde{F}}_{23}$, ${\tilde{F}}_{13}$ and ${\tilde{F}}_{12}$ in the sense that $\partial D/\partial {\tilde{F}}_{23}=\partial D/\partial {\tilde{F}}_{13}=\partial D/\partial {\tilde{F}}_{12}$. There is only one point on it exhibiting this property, which is another unique feature of the failure criterion. If an application of a failure criterion is considered as a way to count the contributions of stresses to the failure of the material, then the formulation of a failure criterion is to count contributions of various coefficients to the failure function. As a rational consideration, coefficients of similar nature should make similar contributions to the characteristics of the failure function once they have been appropriately normalized, so that none of them goes out of step with others. It is therefore perfectly reasonable to postulate that these three coefficients in their normalized form would coordinate themselves somehow in their contributions to the formulation of the failure function. With the normalization as introduced in (2.4) and (2.6), there is one and only one possibility satisfying the above consideration, that delivers (5.12).
(c) Infinite strength is expected as the stresses increase along the longitudinal axis, or along the loading path parallel to the longitudinal axis. In fact, as the stress level increases, the loading paths converge to stress ratio ${\tilde{\sigma }}_{11}:{\tilde{\sigma }}_{22}:{\tilde{\sigma }}_{33}=1:1:1$ as the only stress ratio for the material to exhibit infinite strength. With the anisotropy of the material toned down through the normalization, it is reasonable to expect that the normalized stress ratio to demonstrate infinite strength should be in such an unbiased manner. Any deviation from that would mean a mechanism of some kind that discriminated one or more normalized stress components such that they would make different contributions to failure. In the absence of such a mechanism, an unbiased position will be a natural and also rational position. It should be pointed out that the origin of the coordinate systems is always inside the failure envelope, in general, because the material should not have failed before any stress is applied. Since invariant *A* as defined in (3.3*d*) is non-positive as shown in appendix A, the envelope is always open on the triaxial compression side. In the case of *A* = 0, i.e. when ${\tilde{F}}_{1}+{\tilde{F}}_{2}+{\tilde{F}}_{2}=0$, equation (5.12) produces a cylinder and the envelope opens on the triaxial tension side as well, as assumed in the von Mises criterion.
(d) The quadratic terms on the left-hand side of (5.12) demonstrate full symmetry between each pair of stresses. Symmetry is always attractive as a gift from nature but its presence should not be taken for granted and can only be revealed after the anisotropy of the material has been harmonized. The symmetry is not possible without the elliptic cross-section of the quadric surface turning into a circular one at the same time.

## 6. Integration with special cases for materials of less sophisticated anisotropy

Returning to their dimensional forms, one has

6.1 $$\begin{array}{rl} & F_{23}=-\frac{1}{2}\sqrt{F_{22}F_{33}}=-\frac{1}{2\sqrt{Y_{t}Y_{c}Z_{t}Z_{c}}}\\ & F_{13}=-\frac{1}{2}\sqrt{F_{11}F_{33}}=-\frac{1}{2\sqrt{X_{t}X_{c}Z_{t}Z_{c}}}\\ & F_{12}=-\frac{1}{2}\sqrt{F_{11}F_{22}}=-\frac{1}{2\sqrt{X_{t}X_{c}Y_{t}Y_{c}}}. \end{array}$$

These coefficients to the interactive terms in the failure function have now been obtained mathematically on a rational basis.

With the coefficients to the interactive terms as given in (6.1), the rationalized failure criterion for orthotropic materials can be given as

6.2 $$\begin{array}{r} \left(\frac{1}{X_{t}}-\frac{1}{X_{c}}\right)\sigma_{11}+\left(\frac{1}{Y_{t}}-\frac{1}{Y_{c}}\right)\sigma_{22}+\left(\frac{1}{Z_{t}}-\frac{1}{Z_{c}}\right)\sigma_{33}+\frac{1}{X_{t}X_{c}}\sigma_{11}^{2}+\frac{1}{Y_{t}Y_{c}}\sigma_{22}^{2}+\frac{1}{Z_{t}Z_{c}}\sigma_{33}^{2}\\ -\frac{1}{\sqrt{Y_{t}Y_{c}Z_{t}Z_{c}}}\sigma_{22}\sigma_{33}-\frac{1}{\sqrt{X_{t}X_{c}Z_{t}Z_{c}}}\sigma_{11}\sigma_{33}-\frac{1}{\sqrt{X_{t}X_{c}Y_{t}Y_{c}}}\sigma_{11}\sigma_{22}+\frac{1}{S_{23}^{2}}\tau_{23}^{2}+\frac{1}{S_{13}^{2}}\tau_{13}^{2}+\frac{1}{S_{12}^{2}}\tau_{12}^{2}=1. \end{array}$$

It involves nine independent strength properties altogether, tensile and compressive strengths in each of the principal directions and shear strength in each of the principal planes.

**Figure 3.**
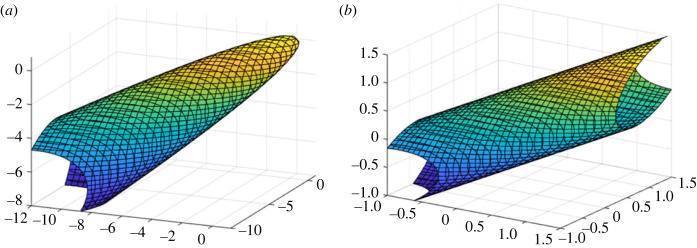
The failure envelope in the subspace of direct stresses. (*a*) An elliptic paraboloid in general or (*b*) an elliptic cylinder when $X_{t}=X_{c}$, $Y_{t}=Y_{c}$ and $Z_{t}=Z_{c}$, simultaneously.

As argued previously, the shear stresses involved in (6.2) moved to the right-hand side of the equation with their contributions shown as a reduction of the value of the right-hand side from 1 to a lower one. Within the subspace of direct stresses, quadric surface (6.2) takes a form of an elliptic paraboloid in general or an elliptic cylinder as a special case when $X_{t}=X_{c}$, $Y_{t}=Y_{c}$ and $Z_{t}=Z_{c}$, simultaneously, as illustrated in figure 3.

Under a hydrostatic stress state, the strengths under tension and compression can be obtained from the following quadratic equation as the two roots respectively, that are both finite:

6.3 $$\begin{array}{rl} & \frac{1}{2}\left({\left(\frac{1}{\sqrt{X_{t}X_{c}}}-\frac{1}{\sqrt{Y_{t}Y_{c}}}\right)}^{2}+{\left(\frac{1}{\sqrt{Y_{t}Y_{c}}}-\frac{1}{\sqrt{Z_{t}Z_{c}}}\right)}^{2}+{\left(\frac{1}{\sqrt{Z_{t}Z_{c}}}-\frac{1}{\sqrt{X_{t}X_{c}}}\right)}^{2}\right)\sigma^{2}\\ & \;-\left(\left(\frac{1}{X_{t}}-\frac{1}{X_{c}}\right)+\left(\frac{1}{Y_{t}}-\frac{1}{Y_{c}}\right)+\left(\frac{1}{Z_{t}}-\frac{1}{Z_{c}}\right)\right)\sigma -1=0. \end{array}$$

The infinite strength can be achieved under only one triaxial compressive stress state of a stress ratio which is defined by the eigenvector ${\left[\begin{array}{ccc} 1/\sqrt{3} & 1/\sqrt{3} & 1/\sqrt{3} \end{array}\right]}^{T}$ of **D** as given in (3.3*c*) corresponding to the eigenvalue 0, i.e.

6.4a $${\tilde{\sigma }}_{11}:{\tilde{\sigma }}_{22}:{\tilde{\sigma }}_{33}=1:1:1,$$

or

6.4b $$\sigma_{11}:\sigma_{22}:\sigma_{33}=\frac{1}{\sqrt{Y_{t}Y_{c}Z_{t}Z_{c}}}:\frac{1}{\sqrt{Z_{t}Z_{c}X_{t}X_{c}}}:\frac{1}{\sqrt{X_{t}X_{c}Y_{t}Y_{c}}}.$$

The material concerned so far has been genuinely orthotropic with different tensile and compressive strengths under a general three-dimensional stress state. Less sophisticated scenarios will be addressed as special cases as follows.

For transversely isotropic materials,

6.5 $$\begin{array}{c} F_{2}=F_{3}\\ F_{22}=F_{33}\\ F_{55}=F_{66} \end{array}\;\mathrm{and}\;\begin{array}{c} F_{23}=-\frac{1}{2}F_{22}\\ F_{13}=F_{12}=-\frac{1}{2}\sqrt{F_{11}F_{22}}. \end{array}$$

Given the relationship of transversely isotropic materials [4–7]

6.6 $$F_{44}=2(F_{22}-F_{23})=3F_{22},$$

the transverse shear strength can be obtained as a consequence of transverse isotropy as

6.7 $$S_{T}=\sqrt{Y_{t}Y_{c}/3}.$$

It means that this particular strength for transverse shear is not an independent property. The so-called fully rationalized Tsai–Wu criterion for transversely isotropic materials [6,7] can be reproduced as a special case of (6.2):

6.8 $$\begin{array}{rl} \left(\frac{1}{X_{t}}-\frac{1}{X_{c}}\right)\sigma_{11}+\left(\frac{1}{Y_{t}}-\frac{1}{Y_{c}}\right)(\sigma_{22}+\sigma_{33})+\frac{1}{X_{t}X_{c}}\sigma_{11}^{2}+\frac{1}{Y_{t}Y_{c}}(\sigma_{22}^{2}+\sigma_{33}^{2}-\sigma_{22}\sigma_{33}+3\tau_{23}^{2})\\ & \;-\frac{1}{\sqrt{X_{t}X_{c}Y_{t}Y_{c}}}\sigma_{11}(\sigma_{22}+\sigma_{33})+\frac{1}{S_{12}^{2}}(\tau_{12}^{2}+\tau_{13}^{2})=1. \end{array}$$

The consistency between the outcomes of these two independent developments helps justify each other. Criterion (6.8) involves five independent strength properties altogether, tensile and compressive strengths in and transverse to the fibre direction and an in-plane shear strength.

For cubically symmetric materials [8], i.e. behaviour of the material is identical in all three principal directions but the shear strength is independent of the tensile or compressive strength, the criterion can be given as another special case of (6.2):

6.9 $$\begin{array}{rl} & \left(\frac{1}{X_{t}}-\frac{1}{X_{c}}\right)(\sigma_{11}+\sigma_{22}+\sigma_{33})+\frac{1}{X_{t}X_{c}}(\sigma_{11}^{2}+\sigma_{22}^{2}+\sigma_{33}^{2}-\sigma_{22}\sigma_{33}-\sigma_{11}\sigma_{33}-\sigma_{11}\sigma_{22})\\ & \;+\frac{1}{S^{2}}(\tau_{23}^{2}+\tau_{13}^{2}+\tau_{12}^{2})=1. \end{array}$$

It involves three independent strength properties, tensile and compressive strengths and a shear strength. In fact, if the failure criterion was formulated for this particular type of materials directly, there would not be any need for the argument as made in the two previous sections. The condition of *D* = 0 would lead to the determination of $F_{23}=F_{13}=F_{12}=-F_{11}/2$, which reproduces the criterion identical to (6.9). This verifies to an extent the rationality of the arguments made in the two previous sections.

For isotropic materials, criterion (6.2) reduces to

6.10 $$\begin{array}{rl} & \left(\frac{1}{X_{t}}-\frac{1}{X_{c}}\right)(\sigma_{11}+\sigma_{22}+\sigma_{33})\\ & \;+\frac{1}{X_{t}X_{c}}(\sigma_{11}^{2}+\sigma_{22}^{2}+\sigma_{33}^{2}-\sigma_{22}\sigma_{33}-\sigma_{11}\sigma_{33}-\sigma_{11}\sigma_{22}+3\tau_{23}^{2}+3\tau_{13}^{2}+3\tau_{12}^{2})=1, \end{array}$$

which reproduces the RCY criterion [22]. It involves two independent strength properties, tensile and compressive strengths.

Assigning a pure shear stress state to the material, the shear strength *S* of the material can be obtained as

6.11 $$S=\sqrt{X_{t}X_{c}/3}.$$

Similarly, each of the above cases applies to materials of equal tensile and compressive strengths simply by dropping the 1st order terms in the failure criterion and replacing the tensile and compressive strengths by a common strength. As an example, when the tensile and compressive strengths *X*_t_ and *X*_c_ become equal, denoted as *X* in (6.4), the von Mises criterion [17] is reproduced as

6.12 $$\sigma_{11}^{2}+\sigma_{22}^{2}+\sigma_{33}^{2}-\sigma_{22}\sigma_{33}-\sigma_{11}\sigma_{33}-\sigma_{11}\sigma_{22}+3\tau_{23}^{2}+3\tau_{13}^{2}+3\tau_{12}^{2}=X^{2}.$$

A well-known outcome of the von Mises yield criterion is that the shear strength *S* of the material is related to the yield strength as follows:

6.13 $$S=\frac{X}{\sqrt{3}}.$$

It is apparently a special case of (6.7) or (6.11) when the material is isotropic with common tensile and compressive strength.

The series of failure criteria as presented above, while as natural consequences of degeneration to less anisotropic materials as special cases, has reproduced some well-known failure criteria in existence. The consistency exhibited should serve as a basic verification of the failure criterion as formulated in the present paper for genuinely orthotropic materials.

For the degree of anisotropy up to orthotropy, there have been established industrial standards to support their measurements. Available strength properties can only be relatively reliably measured from similar tests as established in these standards. Introduction of any further strength properties beyond these without appropriate means to determine them is effectively to allow fudge factors before these properties can be experimentally or rationally determined.

**Figure 4.**
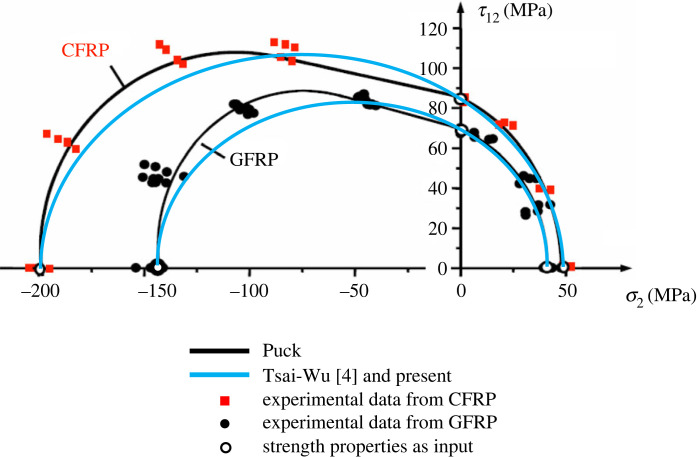
Comparison with available experimental data as well as the predictions from the Puck criterion [2] for the combined in-plane stress states.

As a generally applicable rule, each of the above criteria applies to a two-dimensional stress state (not to be confused with two-dimensional strain state) simply by dropping the relevant terms in the failure criterion. Effectively, this is equivalent to setting stresses not involved in the two-dimensional stress state to zero. As an example, the criterion formulated in this paper has been applied in its special form to two typical types of transversely isotropic composites and compared with available experimental data as well as the predictions from the Puck criterion [2] for the combined in-plane transverse stress and shear stress, as presented in figure 4, where the relevant details including the material properties can be found in [2]. For the materials concerned under the stress states specified, the present criterion reproduces that of the Tsai–Wu criterion, understanding that the conventional Tsai–Wu criterion would lead to anomalies under general three-dimensional stress states before it was fully rationalized [6,7]. It should be pointed out that although the Puck criterion seemed to offer a marginally better fit to the experimental data, it required about a dozen strength properties, the majority of which are not experimentally measurable, and each of the predicted failure loci as shown in figure 4 was composed of three segments of different types of curves. With the present criterion, under a two-dimensional stress condition as was the case concerned, it required only five strength properties, all well known and there were established industrial standards for their experimental measurements. Each of the predicted failure loci according to the present criterion as shown in figure 4 was a single segment of an ellipse. While the present criterion degenerates into a range of well-established criteria consistently for specific categories of materials, such as the fully rationalized Tsai–Wu criterion [6,7] for transversely isotropic materials as elaborated above, the real potential of the present criterion is its applicability to genuinely orthotropic materials for which the Puck criterion is simply inapplicable while the conventional Tsai–Wu criterion tended to leave the coefficients to the interactive terms undetermined.

## 7. The standard form of a quadric surface for the failure criterion

By taking advantage of the standard form of quadric surfaces, the failure criteria for a wide class of materials from genuinely orthotropic to completely isotropic ones can be unified as follows. The failure envelope can be given in one of the two standard forms as follows:

7.1a $$\begin{array}{cccc} \mathrm{Paraboloid}: & s_{22}^{2}+s_{33}^{2}=ps_{11} & \mathrm{if} & {\tilde{F}}_{1}+{\tilde{F}}_{2}+{\tilde{F}}_{2}\neq 0 \end{array}$$

and

7.1b $$\begin{array}{cccc} \mathrm{Cylinder}: & s_{22}^{2}+s_{33}^{2}=c^{2} & \mathrm{if} & {\tilde{F}}_{1}+{\tilde{F}}_{2}+{\tilde{F}}_{2}=0, \end{array}$$

where

7.2a $$p=-\frac{2\sqrt{3}}{9}({\tilde{F}}_{1}+{\tilde{F}}_{2}+{\tilde{F}}_{3}),$$

7.2b $$c=\sqrt{\frac{1}{27}({\left({\tilde{F}}_{2}-{\tilde{F}}_{3}\right)}^{2}+{\left({\tilde{F}}_{1}-{\tilde{F}}_{3}\right)}^{2}+{\left({\tilde{F}}_{1}-{\tilde{F}}_{2}\right)}^{2})+\frac{2}{3}K},$$

7.2c $$\left[\begin{array}{c} s_{11}\\ s_{22}\\ s_{33} \end{array}\right]={\left[T\right]}^{T}\left\{\begin{array}{c} {\tilde{\sigma }}_{11}\\ {\tilde{\sigma }}_{22}\\ {\tilde{\sigma }}_{33} \end{array}\right\}-\left[\begin{array}{c} \delta_{1}\\ \delta_{2}\\ \delta_{3} \end{array}\right]$$

and

7.2d $$[T]=\left[\begin{array}{ccc} t_{1} & t_{2} & t_{3} \end{array}\right]=\left[\begin{array}{ccc} 1/\sqrt{3} & 1/\sqrt{2} & 1/\sqrt{6}\\ 1/\sqrt{3} & 0 & -2/\sqrt{6}\\ 1/\sqrt{3} & -1/\sqrt{2} & 1/\sqrt{6} \end{array}\right],$$

with *t*_1_, *t*_2_ and *t*_3_ being the eigenvectors of **D** corresponding to its eigenvalues 0 and repeated 3/2, respectively.

$$\delta_{1}=\{\begin{array}{cc} \frac{\sqrt{3}}{{\tilde{F}}_{1}+{\tilde{F}}_{2}+{\tilde{F}}_{3}}\left[\frac{1}{18}({\left({\tilde{F}}_{2}-{\tilde{F}}_{3}\right)}^{2}+{\left({\tilde{F}}_{1}-{\tilde{F}}_{3}\right)}^{2}+{\left({\tilde{F}}_{1}-{\tilde{F}}_{2}\right)}^{2})+K\right] & \mathrm{if}\;{\tilde{F}}_{1}+{\tilde{F}}_{2}+{\tilde{F}}_{2}\neq 0\\ \mathrm{Any}\;\mathrm{real}\;\mathrm{value}\;\mathrm{without}\;\mathrm{affecting}\;\mathrm{anything} & \mathrm{if}\;{\tilde{F}}_{1}+{\tilde{F}}_{2}+{\tilde{F}}_{2}=0, \end{array}$$

$$\delta_{2}=-\frac{\sqrt{2}}{6}({\tilde{F}}_{1}-{\tilde{F}}_{3})$$

7.2e $$\mathrm{and}\;\delta_{3}=-\frac{\sqrt{6}}{18}({\tilde{F}}_{1}-2{\tilde{F}}_{2}+{\tilde{F}}_{3}).$$

For brittle materials, compressive strengths are usually higher than tensile ones. Then the values of ${\tilde{F}}_{i}$ (*i* = 1,2,3) tend to be positive. For modern fibre reinforced composites, the trend can be opposite in directions where fibres lie. As long as ${\tilde{F}}_{1}+{\tilde{F}}_{2}+{\tilde{F}}_{3}\neq 0$, the quadric surface as the failure envelope is a circular paraboloid in its standard form. The sense of ${\tilde{F}}_{1}+{\tilde{F}}_{2}+{\tilde{F}}_{3}$ dictates the orientation of the opening of the failure envelope. When ${\tilde{F}}_{1}+{\tilde{F}}_{2}+{\tilde{F}}_{3}>0$, the opening of the envelope faces the negative side of *s*_11_, i.e. the side of triaxial compression in its conventional stress space. A hypothetical position is when ${\tilde{F}}_{1}+{\tilde{F}}_{2}+{\tilde{F}}_{3}<0$. It is a bit uneasy to contemplate the opening on the triaxial tension side which implies an infinite strength under tension. Hopefully, it will not be a commonplace, as the negative value of ${\tilde{F}}_{1}$ can be overridden by positive ${\tilde{F}}_{2}$ and ${\tilde{F}}_{3}$, as in the case of UD composites. An infinite strength here can be a stress level under triaxial stress state at a specific stress ratio, which is significantly higher than the tensile or compressive strength obtained under uniaxial stress. Such a scenario is not actually as exotic as one might perceive as the well-accepted von Mises criterion allows such infinite strength for ductile materials, which leads to the subject of the next scenario.

When ${\tilde{F}}_{1}+{\tilde{F}}_{2}+{\tilde{F}}_{3}=0$, i.e.

7.3 $$\sqrt{\frac{X_{c}}{X_{t}}}-\sqrt{\frac{X_{t}}{X_{c}}}+\sqrt{\frac{Y_{c}}{Y_{t}}}-\sqrt{\frac{Y_{t}}{Y_{c}}}+\sqrt{\frac{Z_{c}}{Z_{t}}}-\sqrt{\frac{Z_{t}}{Z_{c}}}=0,$$

the failure envelope as given in (3.2) is an elliptic cylinder which can then be standardized into a circular cylinder as given in (7.1*b*), of which the von Mises criterion is a special case. Apparently, equal tensile and compressive strengths of a material in all its principal directions is a sufficient condition for an elliptic cylindrical failure envelope, but it is not a necessary condition, in general. In its standard form, value *c* defines the radius of the cylinder, for which a real value can always be obtained. As the contribution from the shear stresses increases, this radius shrinks with the limit given by

7.4 $${\tilde{\tau }}_{23}^{2}+{\tilde{\tau }}_{13}^{2}+{\tilde{\tau }}_{12}^{2}=1+{\left({\tilde{F}}_{2}-{\tilde{F}}_{3}\right)}^{2}+{\left({\tilde{F}}_{1}-{\tilde{F}}_{3}\right)}^{2}+{\left({\tilde{F}}_{1}-{\tilde{F}}_{2}\right)}^{2},$$

which indicates a failure purely by shear stresses.

Although users are unlikely to employ (7.1) as the criterion for their practical failure assessment, the definitive standard form of the failure envelope as defined in (7.1) as a quadric surface is informative. Beyond yield surface for metal plasticity, there has never been such a clear and definitive conclusion in terms of the shape of the failure envelope.

The present criterion has thus vertically integrated a family of different failure criteria for materials of different degree of anisotropy with equal or different tensile and compressive strengths. Such integration would serve at least some kind of assurance of consistency. Unfortunately, there have not been many genuinely orthotropic materials known in the public domain, let alone any systematic test data to validate the criterion at the moment.

As an exception, the obtained criterion may not be applicable to porous materials, such as modern lattice structures, e.g. the lattice structure as shown in figure 1*c* for which an infinite strength under triaxial compression is no longer acceptable. In this case, an ellipsoidal failure envelope as assumed in [14] would be more relevant as reproducing the von Mises criterion is no longer a part of the consideration. It is unfortunately beyond the scope of this paper and a separate development is necessary.

## 8. Discussion

Genuinely orthotropic materials as structural and functional materials are relatively emerging technology, as stated earlier. There is lack of practical applications as well as appropriate criteria to predict their failure under a general three-dimensional stress state. Existing criteria tend to leave gaps or lead to anomalies in their applications. The failure criterion established in this paper is rational in the sense that it relies only on a number of pre-set assumptions, as will be summarized in the next section. These assumptions are mutually independent, individually necessary and collectively sufficient for the failure criterion formulated. The rest of the formulation is purely mathematical and logical manipulations without any ambiguity or inconsistency.

The criterion formulated in this paper is applicable to all materials falling in the broad category of orthotropy under general three-dimensional stresses, including its various special forms, such as for transversely isotropic, cubically symmetric and isotropic materials of different or common tensile and compressive strengths. It can be employed to predict the failure of the material concerned, where failure can be understood differently in different applications, e.g. fracture of the material, onset of plasticity, or deviation from linear elasticity, and in different scales, provided that the material can be effectively considered to be homogeneous. For each of the special case, it reproduces the most well-accepted failure criterion specifically formulated for this type of materials. The actual application of the criterion requires nine strength properties for genuinely orthotropic materials as in (6.2), five for transversely isotropic materials as in (6.8), three for cubic symmetric materials as in (6.9), two for isotropic materials of different tensile and compressive strengths as in (6.8) and one for isotropic materials of common tensile and compressive strength as in (6.12). With the stresses concerned substituted into these equations, respectively, these equations provide the critical conditions for material failure, while a subcritical condition is demonstrated when the right-hand side of these equations produces a value less than 1 and the material is overloaded if a value greater than 1 is produced. It can therefore be incorporated readily in an engineering design code, e.g. any finite-element package.

The major advantage of the criterion formulated is its rationality in the sense that it is always consistent and will never lead to any anomaly and it requires only well-known and experimentally obtainable strength properties. Another advantage of it is its capacity to consistently embrace all most well-accepted failure criteria for simpler materials as special cases. The main disadvantage of the criterion is its failure function being a single quadratic function. As a result, it may not always predict experimental data with the accuracy desired by users. While this is presented as a restriction, any escalation in sophistication tends to introduce strength properties that there is no means to determine appropriately. On many occasions, such properties could only be incorporated as some kind of fudge factors, undermining the rationality of the criterion concerned.

Interested readers are encouraged to help with the full establishment of the criterion formulated by generating necessary experimental strength data, especially those associated with triaxial tensions and compressions and justify the condition for or reveal the restrictions on the assumed infinite strength. In the meantime, with the support of more strength properties available, a more accurate failure criterion might be constructed with a polynomial of a higher order or piecewise defined failure function.

## 9. Conclusion

A quadratic failure criterion applicable to genuinely orthotropic materials has been formulated in this paper, as given in equation (6.2). As the input for a prediction of the failure of an orthotropic material under a given stress state it requires the tensile and compressive strengths of the material in its three principal axes, and shear strengths of the material in its three principal planes, altogether nine strength properties of the material, which can all be measured experimentally and there have been industrial standards to support the respective experimentation.

The coefficients to the interactive terms after appropriate normalization are obtained under a number of pre-set assumptions: (i) the material is homogeneous; (ii) the failure function is a single quadratic function of stresses as given in (2.1); (iii) the failure envelope intersects any coordinate plane defined in terms of any two direct stresses in an ellipse, as implied by condition (2.7); and (iv) the failure envelope must be a real and proper surface other than an ellipsoid, any kind of hyperboloid or hyperbolic paraboloid, which lead to condition *D* = 0.

The reasonableness of normalization introduced can be justified by a special coincidence between the state when the longitudinal axis of the elliptic paraboloidal failure envelope expressed in terms of normalized stresses is inclined at the same angle to all three normalized direct stresses and the state when the cross-section of paraboloid is circular. This can also be described as the symmetry of the failure envelope between normalized direct stresses as far as the quadratic terms are concerned. Another description of the same is an equal sensitivity of the characteristic measure of the failure function to the normalized interactive coefficients, and none of them should make any contribution to the formulation of the criterion disproportionally.

An effort has been made in this paper to transform the quadric failure envelope into its standard form according to analytic geometry. It turns out that it can only be in one of two shapes, an elliptic paraboloid in general or an elliptic cylinder under the strict condition (7.3) with materials of equal tensile and compression strengths as a special case of this kind.

The criterion can be consistently reduced to well-established criteria for materials of reduced degree of anisotropy. They include the rationalized Tsai–Wu criterion for transversely isotropic materials, that for cubically symmetric materials, the well-known RCY criterion and the von Mises criteria for isotropic materials. This fulfils a complete vertical integration of a series of material failure criteria based on a quadratic failure function.

## Data Availability

This article has no additional data.

## Appendix A

With ${\tilde{F}}_{23}$, ${\tilde{F}}_{13}$ and ${\tilde{F}}_{12}$ determined as given in (5.10), it can be shown that

A1 $$A=-\frac{3}{16}{\left({\tilde{F}}_{1}+{\tilde{F}}_{2}+{\tilde{F}}_{3}\right)}^{2}\leq 0.$$

When the less than sign holds in (A 1), the failure envelope defined in (6.2) as a quadric surface will definitely be as an elliptic paraboloidal surface.

The only exception is when

A2 $${\tilde{F}}_{1}+{\tilde{F}}_{2}+{\tilde{F}}_{3}=0,\;\text{i.e.}\;A=0.$$

In this case, it can be proven that the failure envelope degenerates to an elliptic cylinder. As a special case, when $F_{1}=F_{2}=F_{3}=0$, i.e. the tensile and compressive strengths are equal in each principal direction, condition (A 2) is satisfied. For the failure envelope definitely to be an elliptic cylinder, it requires another condition, i.e. $A^{\prime }<0$, as given in (2.7*e*). Under conditions (A 2),

A3 $$A^{\prime }=-\frac{9}{4}K-\frac{3}{8}({\tilde{F}}_{1}^{2}+{\tilde{F}}_{2}^{2}+{\tilde{F}}_{3}^{2}).$$

It should be less than zero in order to fulfil the conditions in (2.7*e*). This can be discussed in two scenarios:

(i) When $F_{1}=F_{2}=F_{3}=0$, for $A^{\prime }<0$, one must have *K* > 0. Given the definition of (3.1), *K* > 0 means that shear stresses should be kept sufficiently low so that they alone should not cause failure. Otherwise, there will not be any real failure envelope in the subspace of direct stresses. In other words, under such shear stresses, the material is no longer able to take any direct stresses.
(ii) When any of $F_{1}$, $F_{2}$ or $F_{3}$ is not equal to zero, ${\tilde{F}}_{1}^{2}+{\tilde{F}}_{2}^{2}+{\tilde{F}}_{3}^{2}\neq 0$. The shear stresses should satisfy
  A4 $${\tilde{\tau }}_{23}^{2}+{\tilde{\tau }}_{13}^{2}+{\tilde{\tau }}_{12}^{2}<1+\frac{1}{6}({\tilde{F}}_{1}^{2}+{\tilde{F}}_{2}^{2}+{\tilde{F}}_{3}^{2}),$$
in order to have a real failure envelope in the subspace of direct stresses, i.e. for the material to be able to sustain some direct stresses.
